# Effect of dynamic neuromuscular stabilization training using the inertial load of water on functional movement and postural sway in middle-aged women: a randomized controlled trial

**DOI:** 10.1186/s12905-024-02972-w

**Published:** 2024-03-04

**Authors:** Shuho Kang, Ilbong Park, Min-Seong Ha

**Affiliations:** 1 Department of Sports Rehabilitation, Busan University of Foreign Studies, 65 Geumsaem-Ro 485Beon-Gil, Geumjeong-Gu, Busan, 46234 Republic of Korea; 2 Laboratory of Sports Conditioning: Nutrition Biochemistry and Neuroscience, Department of Sports Science, College of the Arts and Sports, University of Seoul, 163 Seoulsiripdaero, Dongdaemun-Gu, Seoul, 02504 Republic of Korea

**Keywords:** Dynamic neuromuscular stabilization, Middle-aged women, Functional movement, Postural sway

## Abstract

**Background:**

Chronic stress and diseases occur more frequently in middle-aged compared to younger women and this is often the result of physical, psychological and socio-economic changes. These health consequences reduce lower body muscle mass and flexibility, leading to generalized impairments in functional movement and balance. Dynamic Neuromuscular Stabilization (DNS) training using the inertial load of water is known for its positive impact on functional strength improvement and muscle stabilization.

**Objective:**

This study aimed to determine the effect of DNS training using inertial water loads on functional movement and postural sway in middle-aged women.

**Method:**

A sample of 24 middle-aged women participated in the study and were randomly divided into two groups: the experimental group, *n* = 12 (age: 58.33 ± 1.48 yrs, height: 162.16 ± 1.27 cm, weight: 61.77 ± 2.21 kg) and control group, *n* = 12 (age: 59.58 ± 1.13 yrs, height: 160.1 ± 1.13 cm, weight: 57.51 ± 1.12 kg). Center of Pressure (COP), moving distance, Root Mean Square (RMS), movement area and Functional Movement Screen (FMS) were conducted and analyzed pre- and post-examination. Participants engaged in the DNS training regimen, which utilized the inertial load of water, for 60 min each session, conducted twice weekly for 12 weeks.

**Results:**

There were significant differences in the COP distance (*p* < 0.001), RMS (*p* < 0.05), COP area and FMS test (*p* < 0.001) in the pre-post comparison of each group. And significant differences were found in COP distance (*p* < 0.05), RMS (*p* < 0.05), COP area (*p* < 0.05) and FMS test (*p* < 0.05) between groups. The DNS training improved the dynamic stability of single-leg standing, torso stability and functional movement in middle-aged women.

**Conclusion:**

DNS training programs using the inertial load of water have been shown to be effective in movement improvement and posture retention ability, which is beneficial for functional movement, equilibrium strategy, and dynamic stability of middle-aged women. Furthermore, the DNS training method designed in this study can be useful for trainees who require posture correction in a safe and effective way regardless of their age and gender.

**Supplementary Information:**

The online version contains supplementary material available at 10.1186/s12905-024-02972-w.

## Introduction

Middle age is a period generally characterized by deteriorating physical changes [1], and can also involve exposure to chronic stress, which leads to a higher incidence of chronic diseases [2]. During the transition into menopause, there is a progressive decline in physiological functions, including homeostatic regulation, metabolic efficiency, overall physical condition, and motor skills, which collectively diminish functional flexibility. This decline is part of a negative feedback loop involving the degradation of reaction times, neural conduction rates, and cognitive performance, ultimately complicating the maintenance of basic daily living activities [1]. Physical changes that occur during middle age include a decline in muscle strength [3], and a decrease in proprioceptive function and flexibility [4]. These symptoms are characteristic and can lead to a number of side effects due to impaired balance ability. The loss of balance ability can result in falls due to a lack of neuromuscular coordination [5]. Such falls are not limited to the elderly but become more prominent from the age of 50, highlighting the need for preventive measures [6]. According to a survey conducted by the Centers for Disease Control and Prevention in 2020, injuries caused by falls, especially among women, have been consistently increasing, with a significantly higher rate compared to men [7].

Balance function is closely related to dynamic stability, so the importance of core strength has been well established. Core muscles play a primary role in providing body protection against unexpected perturbations [8]. Core stability factors include intra-abdominal pressure (IAP), spinal compressive forces, and hip and trunk muscle stiffness [9]. In this regard, Dynamic Neuromuscular Stabilization (DNS) training, which utilizes IAP and breathing pattern control, aims to improve trunk stabilization, joint centration for efficient limb movement, and enhancement of neuromuscular coordination [10]. An increase in IAP plays a role in generating pre-tension just before limb movement occurs [11], and core activation is ultimately important for creating smooth limb movements.

Recently, DNS training using the inertial load of water has been utilized as a tool in many training fields to activate core pre-tension by utilizing the hydrodynamic inertia of water flow [12]. This training technique is known to be a method that can enhance core stabilization by increasing muscle activation through rapid changes in the center of gravity (COG) under the influence of gravity [12]. The changes in COG can be measured by displacement of the center of pressure (COP), and the lower the measurement, the better the stability [13]. COP's measured values are used to evaluate stability based on anterior and posterior movement distances, and left and right movement distances, with standard deviation [14] used finally as an indicator of physical stability by showing how much control there is over posture stabilization [15].

Based on previous studies, it can be inferred that DNS exercise improves core stability, joint centration, and neuromuscular coordination in middle-aged women at high risk of falls, thereby reducing the overall risk of falls. Previous studies have reported that DNS exercise improves balance ability [16], but no analysis using COP with more objective measurements has been conducted. Therefore, the purpose of this study is to analyze the effects of a 12-week DNS training program utilizing the inertial load of water on functional movement and postural sway in middle-aged women. We hypothesized that DNS training would lead to improvements in COP and enhancements in FMS.

## Methods

### Participants

The study participants were selected women aged 50 to 65 residing in B metropolitan city, and participants who wished to voluntarily participate were enrolled through a recruitment announcement that came from B University's Lifelong Education Center. The number of participants was determined using G*Power 3.1 Windows program (Kiel University, Kiel, Germany), with the following settings: Tails: two, Effect size f: 0.25, Alpha error probability: 0.05, Power: 0.6. Factoring a 10% dropout rate from the initially calculated 22 participants, a total of 24 participants were recruited upon whom the study was conducted [17]. The subjects of our research were individuals who had not confined themselves to minimal physical activities nor had they dedicated themselves to consistent exercise routines over the previous six months. Furthermore, their inclusion in the study was determined through an intricate process involving responses to a comprehensive health questionnaire, meticulous physical evaluations, and detailed laboratory analyses. The research participants were randomly assigned into two groups: a control group (CON) consisting of 12 participants who maintained their regular daily activities, and an exercise group (EG) consisting of 12 participants who performed DNS training interventions using the inertial load of water for 12 weeks. Participants were assigned to the EG and CON groups in a 1:1 ratio using the block randomization method. The block randomization method of SPSS Statistics software version 27.0 (IBM Corp. Armonk, NY, USA) was used. Since the block size was set arbitrarily by a third-party statistician, all researchers were unaware of the block size and number. The random list was provided in a sealed, non-permeable envelope, and all participants, researchers, and assessors were blinded to the treatment allocation until the end of the study. Homogeneity tests were conducted for both groups. Prior to data collection, the purpose and procedures of the study were adequately explained to the participants and informed consent was obtained from all, prior to any involvement on their part. This study received approval from the Institutional Review Board (IRB) at Busan University of Foreign Studies (7,001,786–202212-HR-001–01) and was conducted in accordance with the Helsinki Declaration [18]. Written signed Informed consent was collected from each participant before the start of the study. This trial was retrospectively registered in the Clinical Research Information Service (CRIS) (Republic of Korea, KCT0008785, 11/09/2023, https://cris.nih.go.kr). The exclusion criteria for the research participants were as follows, and individuals deemed unsuitable in the judgment of the researchers, were excluded from the study. 1) Individuals who rely on equipment for walking and face limitations in physical activities, 2) Individuals who have maintained a regular exercise routine of at least three times a week over the last six months, 3) Individuals suffering from uncontrolled hypertension, 4) Individuals with cardiovascular diseases and musculoskeletal disorders, 5) Individuals who have consumed medications or supplements in the past two months that could potentially impact the study's design, 6) Individuals who have altered their diet and exercise habits, consumed an unbalanced diet, indulged in excessive alcohol consumption, or shown reluctance towards long-term participation. Subsequent to these restrictions, safety matters were additionally verified through medical examinations.The physical characteristics of the participants are depicted in Table 1.

**Table 1:** Characteristics of participants

	Age(yrs)	Height(cm)	Weight(kg)	BMI(kg/m^2^)
Exercise group(*n* = 12)	58.33 ± 1.48	162.16 ± 1.27	61.77 ± 2.21	23.51 ± 0.83
Control group (*n* = 12)	59.58 ± 0.90	160.1 ± 1.13	57.51 ± 1.12	22.70 ± 0.42

Values are presented as mean ± SD. *BMI *Body Mass Index

### Measurements

This study was conducted with a randomized controlled trial and pretest–posttest study design to determine the effect of the DNS training program using inertial water load for 12 weeks. The measurement variables included COP, moving distance, RMS (root mean square), movement area and FMS (functional movement screen). The methods and tools used for measurement are shown in Table 2. For the validation of efficacy, the measurement period was set to coincide with the 12 weeks of DNS training. Participants were instructed to perform 5 min of warm-up and cool-down to ensure peak performance, and sufficient rest was provided between tests. Additionally, all measurements were conducted a total of two times.

**Table 2:** Measurement Devices

Equipment	Model	Manufacture
Motion capture	Oqus 500	Qualisys/ Sweden
Placement measure software	QTM (Qualisys Track Manager)	Qualisys / Sweden
COP analysis software	Visual 3Dv5	C-motion/ USA
COP: Center of Pressure		

#### COP (Center of Pressure)

The distance moved in the anterior–posterior and medial–lateral directions, total distance moved, RMS distance, and movement area (the sum of the areas traversed by the center of pressure) were measured using a force plate (Type 9260 Kistler, Winterthur, Switzerland). The measurement variables were referenced from a prior study [19]. The measurements were conducted with the participants standing on the force plate in a single-leg stance. They started with one knee lifted to a 90-degree angle and performed the RUS (reaching unilateral standing) task. During the performance of the RUS task, non-slip tape was applied to the force plate to prevent slipping, and the participants did not wear shoes. Calibration of the force plate sensors was performed before each measurement to ensure measurement accuracy and consistency.

#### FMS (Functional Movement Screen)

The FMS Kit (Functional Movement Screen Test Kit, USA) was used to assess functional movement. FMS consists of seven movements divided into four categories, and each item is scored on a scale of 0–3, resulting in a total score of 21 [20]. The movement items evaluate balance, core stability, flexibility, and mobility [5]. The potential ability of participants was evaluated to eliminate testing errors resulting from repetitive practice. Therefore, participants were evaluated without prior practice, with only the explanation of the movement methods, allowing for the assessment of their natural movements. To enhance objectivity, the FMS assessment was conducted by three experts who had completed the FMS evaluation training course. The experts included a professor specializing in sports rehabilitation, a sports conditioning coach, and a physical therapist. The measurements were performed by these experts, and the average score from all three was calculated.

### DNS training Program using the inertial load of water

**Fig. 1:**
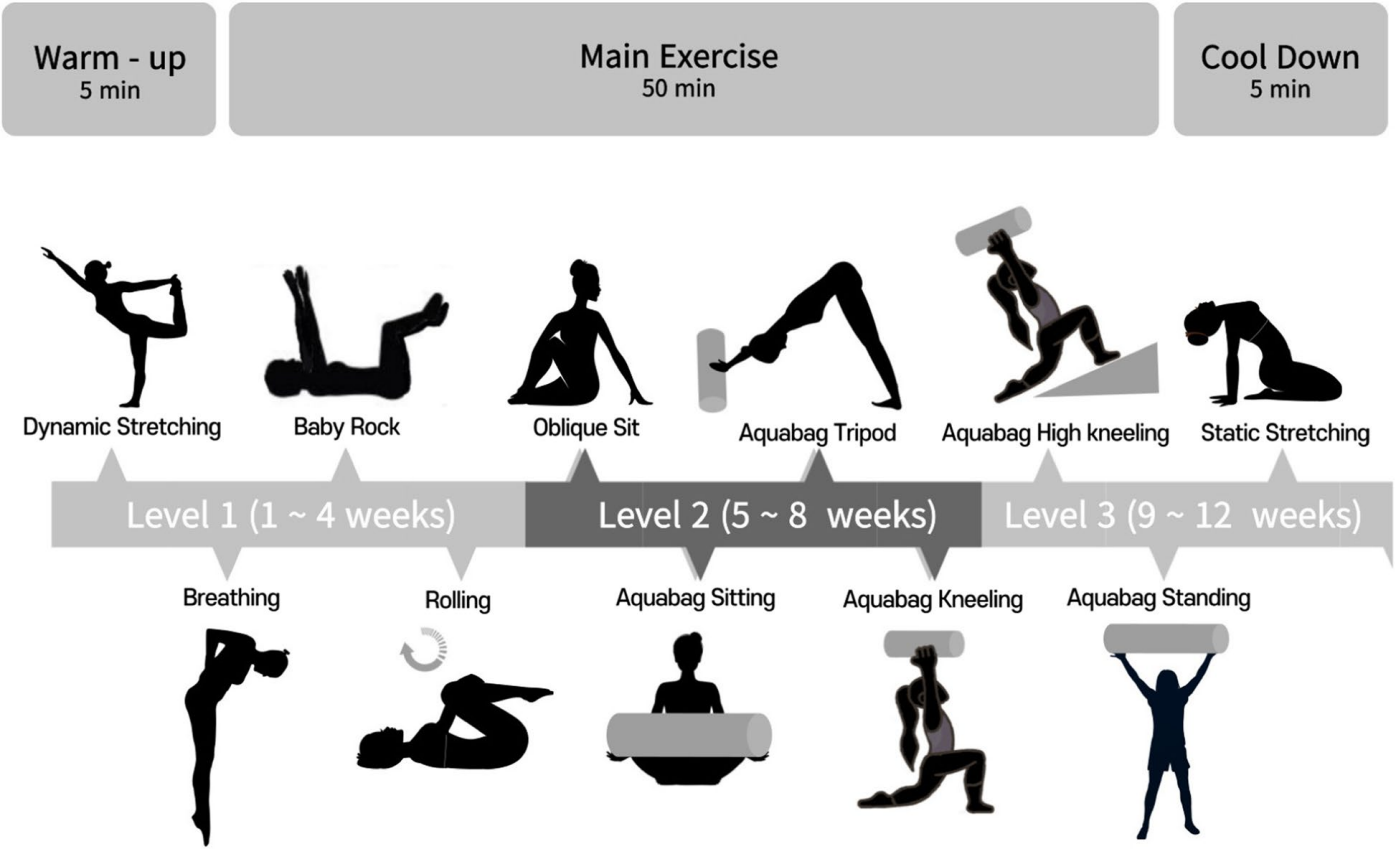
Composition of the DNS training Program using inertial water loads

In this study, the exercise group performed DNS training using the inertial load of water for 60 min per session, twice a week, for a duration of 12 weeks. The DNS Training Program was conducted with the objective of enhancing stabilization through the inertial loads of water. The detailed training program is in Table 3, Fig. 1. The training program was conducted at the same day and time for all participants and was developed by modifying the DNS approach using inertial water loads, based on the research conducted by Mahdieh, Zolaktaf [16]. The inertial loads of water DNS training we conducted was characterized not by evaluating the objectives based on the intensity of the exercise through increasing weights, but rather by assessing balance control using a consistent weight of 5 kg, irrespective of the exercise intensity. The program consisted of a 5-min warm-up, a 50-min main exercise session, and a 5-min cool-down. The warm-up included dynamic stretching, while the cool-down involved static stretching.

**Table 3:** Detailed description of the DNS training Program using inertial water loads

Exercise (Time)	Program	Week	level	Explanation of the Movement
Warm-up (5 min)	Dynamic Stretching			
Main Exercise (50 min)	1.Breathing	1 ~ 4	1	Learning and practicing diaphragmatic breathing at rest (lying, sitting)
		5 ~ 8	2	Practice to Maintain diaphragmatic breathing during maintaining Basic DNS Positions Statically
		9 ~ 12	3	2 + Various movements of single arm or single leg
	2.Baby rock	1 ~ 4	1	Maintaining static movement and focusing on diaphragmatic breathing, Moving both hands at the elbow and shoulder joints in different planes of motion
		5 ~ 8	2	Practice to Maintain diaphragmatic breathing during maintaining Basic DNS Positions Statically
		9 ~ 12	3	Moving one arm and one leg (on the same or opposite side of body) simultaneously in two different planes of motion
	3.Rolling	1 ~ 4	1	Short rolling to the right and left. Being fixed in rolling sideways with short range (three diaphragmatic breathing)
		5 ~ 8	2	1 + Movement of one arm and one leg (on the same or opposite side of body) simultaneously in different planes of motion
		9 ~ 12	3	Combining Rolling and Side Lying
	4. Oblique sit & 5. Aquabag Sitting	1 ~ 4	1	Maintaining static movement and focusing on diaphragmatic breathing Arm movement in different planes of motion
		5 ~ 8	2	1 + lifting the hip and holding it above the ground
		9 ~ 12	3	Combining Oblique Sit and kneeling using aquabag
	6. Aquabag Tripod	1 ~ 4	1	Maintaining static movement and focusing on diaphragmatic breathing Arm and leg movement in different planes of motion
		5 ~ 8	2	1 + Being fixed in Tripod position
		9 ~ 12	3	2 + lifting Aquabag Around the thighs / legs
	7. Aquabag Kneeling & 8. Aquabag High kneeling	1 ~ 4	1	Maintaining static movement and focusing on diaphragmatic breathing Arm movement in different planes of motion
		5 ~ 8	2	Arm movement in different planes of motion using aquabag
		9 ~ 12	3	Knee extension + Arm movement in sagittal plane using aquabag
	9. Aquabag Standing	1 ~ 4	1	Learning and practicing diaphragmatic breathing at standing position
		5 ~ 8	2	Practice to Maintain diaphragmatic breathing during maintaining Basic DNS Positions using aquabag
		9 ~ 12	3	Practice to Maintain diaphragmatic breathing during maintaining DNS Positions using aquabag
Cool-down (5 min)	Static Stretching

### Statistical analysis

**Fig. 2:**
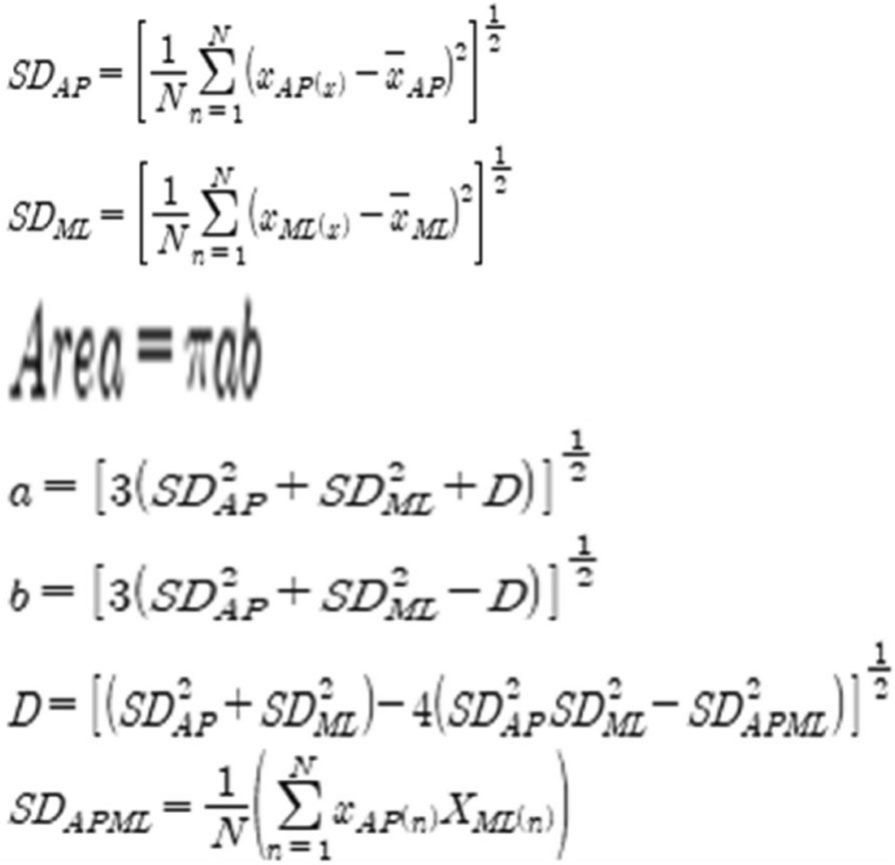
Analysis formula by classifying the COP moving distance

For analysis of COP data, the values obtained through the Qualysis Track Manager 2.2 program were saved as a Microsoft Excel file and calculated in Excel using the following formula. It was calculated by referring to [19], which presented an analysis formula by classifying the COP moving distance into ML (medial—lateral), AP (anterior—posterior), MLAP (medial – lateral—anterior- posterior), distance RMS, and moving area. The formula is as follows Fig. 2. In order to eliminate the initial shaking error that may occur in the experimental environment, the first five seconds of data were discarded [19]. The variables used in the analysis were set as the COP movement distance between coordinates on the x and y axes, the movement area [21], and the RMS of the position value. For all data, the statistical program SPSS for Windows Ver27.0 (IBM Corp. Armonk, NY, USA) was used, and the mean and standard deviation were calculated and filled in. To investigate the effect of 12 weeks of DNS training on COP and FMS, a normality test was first conducted, followed by a 2 × 2 two-way repeated measures ANOVA with treatment and time as independent variables. Post-hoc analysis was performed using LSD, and the statistical significance level was set at *p* < 0.05.

## Results

### Effect of 12 weeks DNS training on COP Distance

**Fig. 3:**
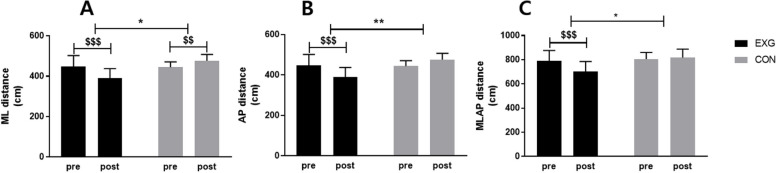
Effect of 12 weeks DNS training on COP Distance in middle-aged women. **A **ML (Medial–Lateral) distance, **B **AP (Anterior–Posterior) distance, **C **MLAP (Medial–Lateral- Anterior–Posterior) distance. Data are presented as the mean ± standard deviation. *p < 0.05; group effects: *p < 0.05, **p < 0.01, $*p* < 0.05; before and after significant: $$*p* < 0.01, $$$*p* < 0.001. EXG: Experimental Group, CON: Control Group

Following 12 weeks of DNS training using inertial water, significant interaction effects on COP variables were found for all of (ML, AP, MLAP) distance, RMS (ML, AP), and COP area. The results are as shown in Table S1, Fig. 3.

First, the ML distance decreased significantly from 448.12 ± 53.91 cm to 390.02 ± 47.50 cm in EXG (*p* < 0.001), and significantly increased from 444.79 ± 26.68 cm to 476.25 ± 31.29 cm in the CON group (*p* < 0.01). Statistically significant differences were found in group (*p* < 0.05) and time*group (*p* < 0.001). AP distance decreased significantly from 553.77 ± 17.48 cm to 491.65 ± 15.76 cm only in EXG (*p* < 0.001). Statistically significant differences were found in time (*p* < 0.5), group (*p* < 0.01), and time*group (*p* < 0.01). The MLAP distance significantly decreased from 789.54 ± 86.32 cm to 703.56 ± 81.81 cm only in EXG (*p* < 0.01). Statistically significant differences were found in time (*p* < 0.01), group (*p* < 0.05), and time*group (*p* < 0.001).

### Effect of 12 weeks DNS training on COP RMS distance, COP Area

**Fig. 4:**
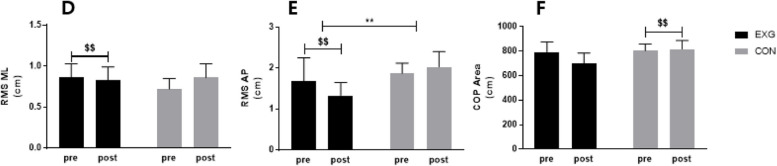
Effect of 12 weeks DNS training on COP RMS distance, COP area in middle-aged women. **D **RMS ML(Medial–Lateral) distance, **E **RMS AP(Anterior–Posterior) distance, **F **Cop Area. Data are presented as mean ± standard deviation. *p < 0.05; group effects: **p < 0.01, $*p* < 0.05; before and after significant: $$*p* < 0.01. EXG: Experimental Group, CON: Control Group

The COP RMS distance and COP area results are shown in Table S1, Fig. 4. RMS ML decreased from 0.86 ± 0.17 cm to 0.72 ± 0.13 cm only in EXG (*p* < 0.01), and statistically significant differences were found in the time*group (*p* < 0.05).

RMS AP significantly decreased from 1.68 ± 0.57 cm to 1.33 ± 0.32 cm only in EXG (*p* < 0.01), and there were statistically significant differences in group (*p* < 0.01) and time*group (*p* < 0.5). The COP area increased significantly from 24.28 ± 8.93 cm to 55.27 ± 55.65 cm only in CON (*p* < 0.1), and a statistically significant difference was found in the time*group (*p* < 0.01).

### Effect of 12 weeks DNS training on FMS

The results of the FMS test are shown in Table 4. Deep squat significantly increased from 1.92 ± 0.67 points to 2.75 ± 0.45 points only in EXG (*p* < 0.05). Statistically significant differences were found in time (*p* < 0.01), group (*p* < 0.05), and time*group (*p* < 0.1). Hurdle step did not show a statistically significant difference between EXG and CON. Inline lunge significantly increased from 1.67 ± 0.65 points to 2.58 ± 0.51 points only in EXG (*p* < 0.001). Statistically significant differences were found in time (*p* < 0.001) and time*group (*p* < 0.01). Shoulder mobility did not show a statistically significant difference in either EXG or CON. Leg raise showed a statistically significant difference in group (*p* < 0.05) and time*group (*p* < 0.05). A statistically significant difference was found only in the Push Up group (*p* < 0.05). Rotary stability did not show a statistically significant difference in either EXG or CON. The total score increased significantly from 13.8 ± 1.53 points to 17.25 ± 1.76 points in EXG (*p* < 0.001). Statistically significant differences were found in time (*p* < 0.001), group (*p* < 0.001), and time*group (*p* < 0.001).

**Table 4:** Effect of 12 weeks DNS training on FMS in middle-aged women

Variables	Group	pre	post	F-value		*p*
Deep Squat (point)	EXG	1.92 ± 0.67	2.75 ± 0.45^$$$^	time	12.922	0.002^**^
				group	5.863	0.024^*^
	CON	1.83 ± 0.58	1.92 ± 0.51			
				time*group	8.650	0.008^**^
Hurdle Step (point)	EXG	1.82 ± 0.60	2.27 ± 0.47	time	2.995	0.098
				group	3.968	0.060
	CON	1.75 ± 0.45	1.83 ± 0.39			
				time*group	1.426	0.246
Inline Lunge (point)	EXG	1.67 ± 0.65	2.58 ± 0.51^$$$^	time	16.851	0.000^***^
				group	0.693	0.414
	CON	1.92 ± 0.51	2.00 ± 0.60			
				time*group	11.702	0.002^**^
Shoulder Mobility (point)	EXG	1.75 ± 0.87	2.00 ± 0.60	time	0.191	0.666
				group	7.755	0.011^*^
	CON	1.33 ± 0.65	1.25 ± 0.62			
				time*group	0.765	0.391
Leg Raise (point)	EXG	2.58 ± 0.51	3.00 ± 0.00	time	2.514	0.127
				group	4.783	0.040^*^
	CON	2.42 ± 0.67	2.33 ± 0.65			
				time*group	5.657	0.026^*^
Push Up (point)	EXG	2.08 ± 0.67	2.58 ± 0.67	time	4.000	0.058
				group	4.508	0.045^*^
	CON	1.83 ± 0.58	2.00 ± 0.60			
				time*group	1.000	0.328
Rotary Stability (point)	EXG	2.00 ± 0.00	2.25 ± 0.62	time	0.512	0.482
				group	3.143	0.090
	CON	1.92 ± 0.51	1.83 ± 0.39			
				time*group	2.047	0.167
Total score (point)	EXG	13.83 ± 1.53	17.25 ± 1.76^$$$^	time	16.206	0.001^***^
				group	26.031	0.000^***^
	CON	13.00 ± 1.48	13.17 ± 1.64			
				time*group	13.331	0.001^***^

Values are presented as mean ± standard deviation. ^*^p < 0.05, ^**^p < 0.01, ^***^p < 0.001, ^$$$^*p* < 0.001, ^$$^*p* < 0.01 vs pre. *EX *Experimental Group, *CON *Control Group, *ML *Medial–Lateral, *AP *Anterior–Posterior

## Discussion

This study hypothesized that a 12-week DNS training program using the inertial load of water would improve COP and FMS scores in middle-aged women. The following results were obtained: DNS training showed significant interaction effects (*p* < 0.001) in all variables of COP Distance, as well as in the variable of COP RMS distance (*p* < 0.05). Furthermore, significant interaction effects were observed in the FMS variables of Deep Squat (*p* < 0.01), Inline Lunge (*p* < 0.01), Leg Raise (*p* < 0.05), and Total score (*p* < 0.001). Furthermore, the DNS training demonstrated significant interaction effects in all variables of COP aiming to maintain a stable posture [15]. In this study, we assessed balance ability using COP variables commonly used in previous research on postural sway. These variables included anterior–posterior and medial–lateral displacement distance, total distance, RMS distance, and sway area (the sum of the areas traversed by the center of mass) [22].

Body balance is achieved through the perception of limb and body movements, as well as head movements, and is influenced by proprioception [23]. Muscle spindles, Golgi tendon organs, and joint receptors are responsible for maintaining balance and providing stability by eliciting responses in the muscle tissue to external perturbations. These proprioceptive receptors respond to external movements and contribute to the preservation of balance and the provision of safety [24].

Based on the FMS measurement results, which assess balance, symmetry, and core stability along with various joint movements, the DNS training demonstrated significant interaction effects in the Deep Squat, Inline Lunge, Leg Raise, and Total Score. Although no significant differences were observed between the two groups in terms of Rotary Stability, a trend towards improvement was noted in those who underwent DNS training compared to those who did not. This too can be considered a very important aspect. This indicates that the 12-week DNS training employed here, improved the body's stability through reduced COP distance, COP area and RMS. The observed changes in FMS are expected to lead to improvements in upright posture and gait ability.

Utilizing unstable surfaces, core and balance exercises that employ the BOSU, Swiss balls and wobble balance boards are commonly used [25]. However, according to a study by Brumagne, Janssens [26], it was found that stimulation of the ankle muscles was actually lower on unstable surfaces compared to rigid and stable surfaces. As well, the effect on COP position changes was minimal. This suggests that during upright posture training on an unstable surface, the ankle angle may not provide accurate information related to the overall body orientation with respect to the surface [27]. To eliminate this concern, we implemented DNS training on a stable surface.

This study found significant interaction effects in the comparison of COP displacement, RMS, and sway area, pre—and post -training. This supports results reported by Adlerton, Moritz [28], where a decrease in COP variables indicated an increase in postural stability. Additionally, it is consistent with the findings of Palmieri, Ingersoll [29], who reported that reduced movement of the center of pressure is associated with the maintenance of postural stability. In particular, by demonstrating that DNS training reduces COP variables and improves balance ability in middle-aged women, this study provides an approach to combat the reported decline in balance capacity and in particular, lateral balance in middle age [3].

Bloomquist, Langberg [30] reported that the Deep Squat and Hurdle Step movements in the FMS assessment can evaluate the coordination between the hip, ankle, and shoulder, since these movements involve vertical gravitational loading on the joints [31] and are related to dynamic stabilization during activities such as upright posture and gait. The mechanics of the Inline Lunge movement are related to lateral balance [32]. Therefore, an increase in the Inline Lunge movement score is interpreted as an improvement in lateral balance ability.

DNS training is a rehabilitation approach developed by Kolar, aimed at developing ideal posture, functional joint centration, optimal breathing, and movement similar to that observed in newborn infants. Park et al. [33] have demonstrated its effectiveness in baseball pitchers with scapular dyskinesis. Additionally, other studies have shown its effectiveness in improving forward head posture and respiration [34] as well as the thickness of neck flexor muscles [35].

Kolar particularly emphasizes the importance of IAP, which is an effective factor in joint centration and efficient muscle activation. Therefore, in this study, a training program was designed to promote spinal alignment, stability, and increased mobility through training in IAP. To generate external perturbations, the exercise involved using the inertial load of water while performing breathing patterns and IAP formation training. The exercise progressed from the tripod position, focusing on proper foot and hand support, to the high kneeling position, which is effective for developing proper weight-bearing ability based on hip joint centration. Horak and Nashner [36] reported that there are changes in postural control strategies and differences in muscle activation patterns based on the location of the center of mass relative to the hip, knee, and ankle joints during external perturbations. In the human body, joints require a fixed point from an ergonomic standpoint for proper mobility, and the core plays an intermediary role in connecting the limbs. Therefore, the function of IAP is considered to be the formation of abdominal stability, enabling the limbs to move freely.

In this study, the Aqua Bag was utilized to incorporate the DNS training and use of inertial water to promote joint centration and prevent joint compression. DNS training using the inertial load of water can enhance core stability and improve overall fitness [12]. Behm, Anderson [37] reported that DNS training increases the number of repetitions capable of being performed during exercise and improves coordination between agonist, antagonist, and stabilizer muscles. This can be considered evidence for improvements in lower limb joint function such as hip joint function, related to maintaining an upright posture, as evaluated in the FMS assessment conducted in this study. DNS training using inertial water load is considered a valid method to activate the inherent potential function of the human body in the process of self-stabilization from external perturbations. Further research related to DNS training using inertial water loads is warranted to explore its potential benefits.

There have been several previous studies that have assessed dynamic stability, but there is limited research that specifically measures COP during the RUS task. The RUS task involves reaching forward with the arm while standing on one leg, and it can be considered a challenging task that requires a high level of coordination. In the human gait cycle, the double-support phase, where both feet are in contact with the ground, only accounts for approximately 10% of the cycle, while the rest consists of the single-support and swing phases [38]. Therefore, in unstable situations, the ability to maintain stability during these phases becomes crucial. When performing purposeful actions, our bodies employ reflexive strategies and compensatory movements to control postural sway and maintain stability [39, 40].

Cresswell, Oddsson [8] emphasized the importance of spinal alignment and stability in the performance of elite athletes in their study. They reported that an increase in IAP contributes to spinal neutrality and stability and highlighted the significant role of co-contraction of muscles such as the transverse abdominal. In this study, the exercise group underwent training to increase IAP and were guided to be conscious of their breathing during movement. The participants in the exercise group maintained their understanding and training of breathing patterns throughout the experimental period. However, the duration of the effects of this training on joint centration was not determined, indicating the need for further research in this area. Additionally, the study focused on measuring balance ability through variables such as COP and FMS. It is suggested that future research should involve measuring electromyography, which directly reflects muscle responses.

## Conclusion

The study concludes that the training program effectively enhanced balance-related factors. Postural sway indicators, including COP distance, RMS, and movement area, showed improved stability through reduced distances. FMS scores for exercises like deep squats, hurdle steps, and inline lunges, which assess movement efficiency and are crucial for upright posture and gait, significantly improved. These findings suggest the program's efficacy in boosting balance by enhancing hip joint stability and mobility, as well as stability in the knee, ankle, and trunk.

### Supplementary Information

**Supplementary Material 1.**

## Data Availability

All data generated or analyzed during this study are presented in the manuscript. Please contact the corresponding authors for access to the data presented in this study.
