# Supplementary material for: Effect of dynamic neuromuscular stabilization training using the inertial load of water on functional movement and postural sway in middle-aged women: a randomized controlled trial
Source: BMC Womens Health. 2024 Mar 4;24:154. doi: 10.1186/s12905-024-02972-w (PMC10910739; doi:10.1186/s12905-024-02972-w)
Supplement: Supplementary file 1 — Supplementary Material 1. [file 12905_2024_2972_MOESM1_ESM.docx]

| Table S1. Effect of DNS training on COP displacement in middle-aged women. | | | | | | |
| --- | --- | --- | --- | --- | --- | --- |
| Variables | Group | pre | post | F-value | | *p* |
| ML distance (cm) | EXG | 448.12±53.91 | 390.02±47.50^$$$^ | time | 4.089 | 0.055 |
|  |  |  |  | group | 7.095 | 0.014^*^ |
|  | CON | 444.79±26.68 | 476.25±31.29^$$^ |  |  |  |
|  |  |  |  | time*group | 46.224 | 0.001^***^ |
| AP distance (cm) | EXG | 553.77±60.55 | 491.65±54.60^$$$^ | time | 5.795 | 0.025^*^ |
|  |  |  |  | group | 8.155 | 0.009^**^ |
|  | CON | 570.53±39.06 | 581.89±54.02 |  |  |  |
|  |  |  |  | time*group | 12.138 | 0.002^**^ |
| MLAP distance  (cm) | EXG | 789.54±86.32 | 703.56±81.81^$$$^ | time | 9.330 | 0.006^**^ |
|  |  |  |  | group | 5.311 | 0.031^*^ |
|  | CON | 804.19±56.34 | 817.95±69.61 |  |  |  |
|  |  |  |  | time*group | 17.802 | 0.001^***^ |
| RMS ML  (cm) | EXG | 0.86±0.17 | 0.72±0.13^$$^ | time | 2.328 | 0.141 |
|  |  |  |  | group | 1.164 | 0.292 |
|  | CON | 0.83±0.16 | 0.86±0.17 |  |  |  |
|  |  |  |  | time*group | 4.562 | 0.044^*^ |
| RMS AP  (cm) | EXG | 1.68±0.57 | 1.33±0.32^$$^ | time | 1.032 | 0.321 |
|  |  |  |  | group | 11.247 | 0.003^**^ |
|  | CON | 1.87±0.25 | 2.03±0.37 |  |  |  |
|  |  |  |  | time*group | 7.090 | 0.014^*^ |
| COP Area  (cm) | EXG | 32.56±20.21 | 15.68±7.21 | time | 0.869 | 0.361 |
|  |  |  |  | group | 2.601 | 0.121 |
|  | CON | 24.28±8.93 | 55.27±55.65^$$^ |  |  |  |
|  |  |  |  | time*group | 10.001 | 0.005^**^ |
| Values are presented as mean±standard deviation. ^*^p<0.05, ^**^p<0.01, ^***^p<0.001, ^$$$^p<0.001, ^$$^p<0.01 vs pre. | | | | | | |
| EXG: Experimental Group, CON: Control Group, ML: Medial-Lateral, AP: Anterior-Posterior | | | | | | |
